# Seroprevalence of leptospirosis in a Mexican military population working with animals

**DOI:** 10.1002/puh2.193

**Published:** 2024-05-29

**Authors:** Juan Ramon Ayala‐Torres, María Fernanda Hernández‐Morales, Valeria María Alanis‐Gallardo, Laura Olivia Arvizu‐Tovar, Orbelin Soberanis‐Ramos

**Affiliations:** ^1^ Departamento de Salud Pública y Medicina Preventiva, Facultad de Medicina Veterinaria y Zootecnia Universidad Nacional Autónoma de México México City México

**Keywords:** leptospirosis, military population, occupational risk, seroprevalence

## Abstract

**Background:**

Leptospirosis is a zoonotic disease and a challenge to global public health. There is an occupational risk, particularly in populations with direct contact with animals and in high‐humidity environments, which favors the survival of leptospires. This study aimed to determine the seroprevalence of leptospirosis in military personnel working in close contact with animals in México and to describe the available preventive measures and protection levels.

**Methods:**

A cross‐sectional study was conducted from March to October 2015. Information regarding protective factors in daily activities was gathered through a self‐evaluation questionnaire. The serum samples of participants were analyzed through enzyme‐linked immunoassay (ELISA) and microscopic agglutination test.

**Results:**

Serums were obtained from 65 active military personnel, 56 males (86.2%) and 9 females (13.8%). Out of the total, 54 (83.1%) tested positive for infection by leptospirosis, 49 were males (87.5%) and 5 were females (55.6%). The highest seroprevalence age group was in the ≥45 years group (15, 23.07%), where all tested positive. Regarding military ranks, 100% of the highest hierarchy turned out positive: Officers (4 out of 4) and Chiefs (14 out of 14); and troops resulted in a seroprevalence of 76.5%. Protection equipment available during daily chores included: Overall, 64.6% had gloves and 53.8% had boots; the reported frequency for the use of gloves was 35.3% (46/65) if worn during more than half of the workday, yet 29.2% (19/65) reported never wearing them.

**Conclusions:**

This study makes the petition to implement protocols of continuous training regarding labor risks and having an epidemiologic surveillance program for exposed personnel indispensable to improve the health and sanitary conditions of military personnel who work in direct contact with animals.

## INTRODUCTION

Leptospirosis is a neglected and reemerging disease of global public health importance and one of the main zoonotic diseases worldwide. This is because of the high morbidity and mortality both in humans and animals, particularly in tropical and subtropical zones. It is estimated that the highest morbidity and mortality results in resource‐limited countries, where it causes an important negative impact on human welfare, with an estimated one million cases and approximately 59,000 deaths each year; it has been identified that the majority of the referred regions are highly neglected and/or have low monitoring [1]. Employment is a risk factor for people, due to direct contact with infected animals and their urine, and contact with contaminated water and soil, conditions that are responsible for most of the infections in farmers, veterinaries, slaughterhouse workers, and meat inspectors [2]. In México, a range from 3.2% to 15.6% of seroprevalence has been reported in rural populations [3, 4, 5] and up to 18.2% in slaughterhouse workers [6].

Currently, the military population is viewed as highly vulnerable to this illness due to the execution of operations and activities that bring them in direct contact with reservoirs or, in fact, expose them to diverse conditions that increase the risk of infection, such as the deployment of troops in the case of severe flooding. There has been reported seroprevalence in American, Korean, and Malaysian military populations of 11.6%, 10.1%, and 16.2%, respectively [7, 8, 9].

Among daily chores performed by military personnel in México, those that imply direct contact with different types of animals are military K‐9 units during the care of canines assigned to the diverse units and operations (detection of explosives or narcotics), horses employed in activities of Armored Calvary and its breeding and attention management, exotic species found in capture and search operations, and small companion animals during support tasks in the face of emergencies caused by disasters or catastrophes where animals are at risk. Nevertheless, in México, military occupation risk due to working outdoors and handling animals has not been reported. The purpose of this study was (1) to determine the seroprevalence of leptospirosis in military personnel working in close contact with animals in México and (2) to describe the protection level of such a population.

## METHODS

### Study design and study site

A cross‐sectional study was conducted at the military zone in México City (SEDENA Campo Militar 1‐A) and carried out on a military population involved with routinely handling animals from March through October 2015. The average temperature in this zone is 19.9°C (68°F), with a minimum of 13.2°C (55.7°F) and a maximum of 26.7°C (80°F). The potential *Leptospira* spp. reservoirs found in this area are mainly free‐ranging urban dogs.

### Study population

The selection criteria included the following: to be serving staff, to know how to read and write, and to have been in occupational risk. The total number of military personnel within this criteria was 128 in the México City unit. The recruitment of participants was carried out in a voluntary, anonymous manner for research purposes, for which they signed their informed consent before questionnaire application and blood sample collection.

### Sample size and sampling method

Out of a total of 70 people in service who worked with animals, 65 agreed to participate, out of which 56 (86%) were males and 9 (14%) were females. Subsequently, in the same place of recruitment, a blood sample was taken through venipuncture following the WHO Guidelines on Drawing Blood (Geneva, 2010). These samples were kept at 4°C in a Specimen Transport Cooler during shipping (no more than 1 h after the sample was taken) and reception at the Public Health Laboratory of the Military Graduated School in Health Services and the Faculty of Chemistry of the Universidad Nacional Autónoma de México.

The next step was to carry out a test for each serum through a commercial enzyme‐linked immunoadsorption test kit (ELISA‐IgM) (Standard Diagnostics). In the case of serums that exhibited antibodies against *Leptospira* spp., a microscopic agglutination test (MAT) against contra *Leptospira interrogans* was performed, according to the diagnostic manual by the Epidemiologic Diagnostic and Reference Institute (InDRE per its Spanish acronym). Serums with an optic density value greater than or equal to 1:80 were regarded as positive/evidence of *Leptospira* spp. infection.

### Data collection

Data gathering was carried out using a questionnaire previously designed and used by researchers and subsequently validated by an Internal Committee; in addition, a pilot trial through the Internet was carried out with 39 participants. This questionnaire was designed to be self‐applied and included questions regarding gender, age, education level, military raking, housing conditions, recorded symptoms compatible with a condition of leptospirosis, handling of animals during professional work labor, and participating in activities regarded as high‐risk.

### Data entry and analysis

Statistical analysis was carried out using SPSS V.21 (IBM Corp. Released 2012. IBM SPSS Statistics for Windows, Version 21.0: IBM Corp.). A descriptive analysis of the variables of interest was performed, using frequencies and proportions. A bivariate analysis was performed with the Chi‐square (*χ* ^2^) association test. The variable of interest was the seropositivity (yes vs. no). The independent variables were: gender (male, female), age group <29 years, 30–34 years, >45 years), educational level (basic, undergraduate, and graduate), military ranking (Troop, Chief, and Official), availability of gloves (yes and no), hand‐washing (yes and no), and prior cleaning (yes and no). Results were expressed as frequencies and percentages, and variables were measured through *χ* ^2^, with a 95% confidence interval. Statistical significance was regarded, if the probability value was equal to or lower than 0.05.

### Ethics approval

The design of the study was presented and accepted by the Military Graduated School in Health Services Committee to comply with the Helsinki Declaration (World Medical Association, 2014).

## RESULTS

### Population characteristics

Regarding age, the most common was found in the range of 30–44 years, with 27 (41.5%) participants, followed by the 18–29 age group with 23 (35.4%) participants. Regarding participants’ highest education level, 41.5% (27) were basic (9 years of schooling), 41.5% (27) were superior (17 years of schooling), and 16.9% (11) were middle (12 years of schooling). Regarding raking, 72.3% (47) informed that their military Rank was that of the troop, followed by 21.5% (14) as Chiefs and 6.2% (4) as Officials.

### Seroprevalence analysis

As far as the ELISA test, 54 (83.1%) participants tested positive and 11 (16.9%) tested negative, out of which 54 refer to 5 out of 9 female participants (55.5%); and to 49 of the 56 male participants (87.5%). Seroprevalence found by age range was 56.5% in the ≤29 years group, 96.5% in the 30–44 years group, and 100% in the group of ≥45 years. Participants whose academic level was basic and superior had a seroprevalence of 85.1% and 88.8%, respectively (Table 1).

**TABLE 1:** Distribution of sociodemographic characteristics for leptospirosis seroprevalence of Mexico City's military personnel from March to October 2015.

Characteristic	Number of participants	ELISA positivity *n* (%)	*p* Value
Gender			
Female	9 (13.8)	5 (55.5)	0.04
Male	56 (86.1)	49 (87.5)	
Age group (years)			
≤29	23 (35.3)	13 (56.5)	
30–44	27 (41.5)	26 (96.2)	
≥45	15 (23.0)	15 (100)	
Education level			
Basic	27 (41.5)	23 (85.1)	0.15
Undergraduate	11 (16.9)	7 (63.6)	
Graduate	27 (41.5)	24 (88.8)	
Military ranking			
Troop	47 (72.3)	36 (76.5)	
Chief	14 (21.5)	14 (100)	
Official	4 (6.1)	4 (100)	
Availability of gloves			
Yes	42 (64.6)	36 (55.3)	0.46
No	26 (40)	18 (27.6)	
Hand washing			
Yes	23 (35.5)	19 (29.2)	0.53
No	41 (63.0)	35 (53.8)	
Prior cleaning			
Yes	29 (44.6)	23 (35.3)	0.48
No	36 (55.3)	31 (47.6)	

Abbreviation: ELISA, enzyme‐linked immunoassay.

### Availability and usage of protective measures

Participants describe handling practices in animals during their professional activities, out of which 83.1% (54) indicated having some type of protection during their handling of animals (mainly horses). Equipment and materials available and most widely used during activities were gloves and boots and/or shoe protectors with 64.6% and 53.8%, respectively. The reported regularity for the use of gloves was that 15.4% did wear them all the time, and 29.2% reported never using them (Table 2).

**TABLE 2:** Availability and usage of protective measures of Mexico City's veterinary service and remount military personnel from March to October 2015.

	Availability *n* (%)		Use frequency during labor activities *n* (%)		
Protective gear	Yes	No	Never	Less than half of the time	More than half of the time
Gloves	42 (64.6)	23 (35.4)	19 (29.2)	23 (35.3)	23 (35.3)
Face mask	26 (40)	39 (60)	38 (58.4)	19 (29.2)	8 (12.3)
Apron	26 (40)	39 (60)	37 (56.9)	16 (24.6)	12 (18.4)
Boots	35 (53.8)	30 (46.2)	33 (50.7)	15 (23.0)	17 (26.1)
Others	54 (83.1)	11 (16.9)	43 (66.1)	4 (6.1)	12 (18.4)

## DISCUSSION

This is the first study describing the seroprevalence of leptospirosis in Mexican military personnel working directly with animals. The seroprevalence of leptospirosis was 83.1%, where men displayed a seroprevalence of 87.5%, whereas women a 55.6%. The highest seroprevalence age group was ≤45 years (100%). In terms of military rank, 100% of Officials (4) and Chiefs (14) tested positive for infection, whereas in Troops, a seroprevalence of 76.5% was identified. Data obtained showed that the most severely affected participants were those between 30 and 40 years old (48.1%), with a Troop rank of 72.3%.

In this study, seroprevalence was revealed to be greater than that described in other military populations, such as the US military with 4% [10], 16.2% in Malaysia [8], and 8% in Nepal [11], although these studies do not state if their population was working directly with animals. We can compare these results to those of another Mexican population working directly with animals or animal‐related products. For example, it was stated that meat workers with rural residences had a more than twofold higher seroprevalence (37.5%) of *Leptospira* infection than the one reported in the general population in rural Durango (north of México) [6], with a particular statement that work (raising animals and agriculture) was a risk factor for *Leptospira* seropositivity. This high occupational risk was also stated in Mexican dairy farm workers, where a study found that the seropositivity of anti‐*Leptospira* antibodies among the population was 46.8% [12] and that 88% of slaughterhouse workers tested were also seropositive. Is worth mentioning that they employed MAT as a confirmatory test for illness, the cut‐off point is not standardized, which could explain differences in magnitude, and used a cut‐off point of ≥100, thus seroprevalence could be subestimated if using a higher cut‐off point, the reason for which the use of ≥80 was determined. It has been reported that the diagnostic value of MAT will depend on the cut‐off point, according to the seroprevalence level exhibited in the regions (high or low) and the stage of the illness, with the best cut‐off being that of 1:50 in low seroprevalence regions and 1:200 for regions with a high seroprevalence [13].

Men had a seroprevalence of 75% compared with 55%, although it is worth mentioning that 90% of this military population belonged to the male category, which could represent gender bias. A study carried out in the military population of the United States showed that the percentage of seroconversion in women was significantly greater than in men, which suggests that they could be at higher risk of leptospirosis [14]. Historical subrepresentation of women in the army forces could be a factor of the poor knowledge regarding the prevalence and manifestations of the disorder in this work activity, particularly if there are exposition differences in terms of chores and assignments within the workplace. Occupations in which there are differences between men and women would require precise primary prevention strategies that take into account such differences and have significant social and public health implications [15, 16].

It can be seen that the highest percentage of seroprevalence is found in the 30–44 age range, with 48.1%, which can be explained because it is the age group where the majority of personnel are in service, but greater than that reported in other military populations, where the risk factor turned out to be in the 18–30‐year age range in the EE.UU. and Guam [7, 10], or even in those that had less than 6 years in active service in the military personnel of Korea [9].

On the other hand, for Military Ranking, troops exhibited a seroprevalence of 76.5%, whereas Chiefs and Officials had 100%. The result differs from that reported in another study of militaries in Malaysia, where officials were less affected by *Leptospira* spp. [8]. The explanation is that this study is focused on a military population that worked in close contact with animals, where the highest rankings are held by veterinarians, and one of their duties is prevention and prophylaxis of livestock diseases and other animals used in the army; thus, they represent the most vulnerable group of this zoonotic disease.

It was found that in some protective gear cases, even though there is a middle–high percentage availability, such as gloves at 64.4%, there is a high risk of infection; in the same case of gloves, there is a 64.6% high‐risk, which translates that their level of protection is low; this can be since even though such materials are available for their protection, they are not used correctly or are simply not used.

One of the strengths of this study is that it shows seroprevalence data in a military population that are in direct contact with animals, which implies a significant risk of infection. One limitation of the study was the military security clearance. The time to obtain authorization that allowed us to use data publicly was extended (7 years), due to the privacy and national security risks of this data. Likewise, the main weakness is leptospirosis subdiagnostic due to the military personnel's low capacity to diagnose effectively and timely, thus the possibility that some of the participants have had the illness but did not have a correct diagnosis.

This study can provide baseline evidence of failure to effectively prevent the disease of leptospirosis in the military population. Therefore, it shows a need to establish awareness programs by public health experts (health professionals and veterinarians) to emphasize the implementation of preventive practices in daily military tasks. In addition, the continuous implementation and evaluation of these measures will assess the impact of interventions to mitigate the occupational risk factors of the disease.

## CONCLUSIONS

The seroprevalence of leptospirosis among military personnel who work in close contact with animals in México City is high. There is a need to create and implement training programs regularly regarding zoonotic illness and implementation of epidemiologic surveillance of personnel exposed to it to improve health and work conditions in military populations.

## AUTHOR CONTRIBUTIONS

*Conceptualization; methodology*: Ayala‐Torres Juan Ramon and Soberanis‐Ramos Orbelin. *Writing—original draft preparation; writing—review and editing*: Ayala‐Torres Juan Ramon, Hernández‐Morales María Fernanda, Arvizu‐Tovar Laura Olivia, Alanis‐Gallardo Valeria María, and Soberanis‐Ramos Orbelin. All authors have read and agreed to the published version of the manuscript.

## CONFLICT OF INTEREST STATEMENT

The authors certify that they have no affiliations with or involvement in any organization or entity with any financial or non‐financial interest in the subject matter or materials discussed in this manuscript.

## ETHICS STATEMENT

The design of the study was approved by the Military Graduated School in Health Services Committee, including the Bioethics Committee (OF/EMGS/SA/141101/MSP/01/PIJR01) to comply with the Helsinki Declaration (World Medical Association, 2013). All study participants provided signed informed consent in the local language (Spanish) before data and sample collection.

## PATIENT CONSENT STATEMENT

The recruitment of participants was voluntary and anonymous for research purposes, for which they signed their informed consent.

## Data Availability

The data supporting this study's findings are available from the corresponding author upon reasonable request.
